# Evaluation of onset of pain relief from micronized aspirin in a dental pain model

**DOI:** 10.1007/s10787-012-0121-0

**Published:** 2012-01-29

**Authors:** S. A. Cooper, M. Voelker

**Affiliations:** 1 Clinical Trial Consultant, Palm Beach Gardens, FL USA; 2 Bayer Consumer Care, Morristown, NJ USA; 3 Bayer HealthCare, Building K56, 51368 Leverkusen, Germany

**Keywords:** Aspirin, Acetylsalicylic acid, Onset of action, Dental pain, Acute pain

## Abstract

A new formulation of a micronized acetylsalicylic acid swallowable tablet with an effervescent component (FR-aspirin) was evaluated in two independent studies using the dental impaction pain model. These clinical studies were performed to confirm the results of preclinical dissolution studies and human pharmacokinetic studies, which indicated an improved onset of analgesia without compromising duration of effect or safety. Study 1 evaluated a 650-mg dose of aspirin and Study 2 evaluated a 1,000-mg dose of aspirin. Both studies were double-blinded, parallel group and compared to regular aspirin (R-aspirin) and placebo. Speed of onset was measured by the double stopwatch method for time to both first perceptible relief and meaningful relief. In both studies, the FR-aspirin was significantly faster (*p* < 0.038–0.001) than both R-aspirin and placebo for both onset measures. There were no significant differences between FR-aspirin and R-aspirin for peak or total effects and both treatments were significantly better than placebo. For first perceptible relief, FR-aspirin onset was 19.8 and 16.3 min for 650 mg and 1,000 mg, respectively, compared to 23.7 and 20.0 for R-aspirin. For meaningful relief, FR-aspirin onset was 48.9 and 49.4 min for 650 mg and 1,000 mg, respectively, compared to 119.2 and 99.2 for R-aspirin. These efficacy studies clearly demonstrate that the onset of analgesic efficacy is dramatically improved by adding an effervescent component and micronized active ingredient to the swallowable tablet aspirin formulation. The enhanced onset did not adversely impact either the peak effect or duration of effect or tolerability compared to regular aspirin.

## Introduction

The two efficacy studies described in this paper were conducted to evaluate the speed of relief onset from a new aspirin swallowable tablet formulation compared to regular aspirin. Based on the improved dissolution and pharmacokinetic profile, the expectation was that this formulation would have a clinically meaningful advantage in time to onset of pain relief.

Self-treatment of acute mild-to-moderate pain with over-the-counter (OTC) analgesics is well established in the general population. Aspirin (acetylsalicylic acid, ASA) is one of the most commonly used analgesics worldwide for this purpose. Its efficacy has been shown in several acute pain conditions, including tension-type headache (Steiner et al. 2003; Martinez-Martin et al. 2001; MacEachern et al. 2002), migraine headache (MacGregor et al. 2002; Lange et al. 2000; Diener et al. 2004a, b, 2006; Lipton et al. 2005; Lampl et al. 2007), sore throat (Eccles et al. 2003), primary dysmenorrhea (Zhang and Li Wan Po 1998) and dental pain (Forbes et al. 1991, 1992; Cooper 1981a, b), as well as fever (Bachert et al. 2005).

The key domains recognized by consumers for an efficacious acute analgesic are fast onset, long duration and complete pain relief. Fast onset of action is of particular importance in an outpatient, self-medication environment. Several pain models have been performed to assess pain relief and onset of relief (Cooper 1983); however, the Dental Impaction Pain Model (DIPM) is the most well established. The sensitivity, reproducibility and reliability of this model are well documented (Cooper and Desjardins 2010; Desjardins et al. 2002). The American Food and Drug Administration and the European Medicines Agency both identified the third molar extraction dental pain model as acceptable for assessing analgesia in acute mild-to-moderate pain (Food and Drug Administration 1977; European Medicines Agency 2002).

A novel aspirin formulation with an effervescent component consisting of sodium carbonate and micronized active ingredient has been developed. This new swallowable tablet formulation has been shown to decrease substantially the in vitro dissolution time and in vivo time to maximum plasma concentration The small active ingredient particle size provides a much larger surface area than the Aspirin^®^ regular tablet resulting in faster dissolution and consequently faster in vivo absorption (Voelker 2011).

Two tablet strengths of the new formulation fast release (FR-aspirin) contain either 325 or 500 mg aspirin, resulting in a total dose of either 650 or 1,000 mg. In these two efficacy studies, we investigated whether the pharmaceutical and pharmacokinetic properties were associated with a faster onset of pain relief compared to a corresponding strength of regular Aspirin^®^ 325 mg or 500 mg (R-aspirin).

## Methods

The studies were designed to evaluate the analgesic efficacy of a single, oral dose of FR-aspirin tablets compared to R-aspirin tablets and placebo in subjects with postsurgical pain resulting from the surgical removal of impacted third molars. Patients were required to have one of the following surgical procedures: either two partial bony impacted mandibular third molars, one full bony impacted mandibular third molar, or one partial bony impacted and one fully bony impacted mandibular third molars removed. Additionally, any maxillary third molars could have been removed regardless of impaction level.

Each study was conducted at a single study site. For Study 1, the treatment arms were: placebo, R-aspirin 1,000 mg, FR-aspirin 1,000 mg. For Study 2, the treatment arms were: placebo, R-aspirin 650 mg, FR-aspirin 650 mg. Both were randomized, double-blind, parallel group and single-dose studies. The randomization scheme in both studies was 2:2:1 for the ratio of active drugs to placebo. Because the FR-aspirin and R-aspirin tablets were not identical in appearance, subjects had a blinding device applied and the study drugs were administered by a third party who was not otherwise involved in the conduct of the study. The aspirin comparator was commercially available Aspirin^®^ regular tablet 500 mg or Bayer Aspirin^®^ 325 mg tablets (both Bayer HealthCare, Germany).

Studies were conducted according to the principles of the International Conference on Harmonisation Harmonised Tripartite Guideline E6(R1): Good Clinical Practice (GCP), the World Medical Association Declaration of Helsinki and its most recent amendments, and United States Title 21 of the Code of Federal Regulations Parts 50 and 56 concerning informed consent and IRB regulations. Written informed consent was obtained from each subject at screening before the initiation of any study-related procedures.

Patients were included in the studies if they had moderate or severe postsurgical pain within 1–4 h after surgery and a score of ≥5 on the 11-point numerical pain intensity rating scale (NRS) (0 = no pain to 10 = very painful).

Patients were provided with two stopwatches starting at study drug administration: the first was stopped when they first felt any perceptible pain relief and the second was pressed when they felt pain relief that was in their estimation meaningful. The patients recorded pain intensity (PI) and pain relief (PR) at 10, 20, 30, 40, 50 and 60 min and at 2, 3, 4, 5 and 6 h after dosing. PI was rated on a four-point categorical pain intensity scale (0 = no pain, 1 = mild pain, 2 = moderate pain, 3 = severe pain); PR was rated using a five-point categorical pain relief rating scale (0 = no relief, 1 = a little relief, 2 = some relief, 3 = a lot of relief, and 4 = complete relief). At the 6-h observation or at the time of rescue analgesic if it occurred prior to the 6th h observation, subjects completed a five-point categorical global evaluation of the study drug (0 = poor, 1 = fair, 2 = good, 3 = very good, and 4 = excellent). Rescue medication was Lortab 5 (hydrocodone 5 mg/acetaminophen 500 mg) tablets. Adverse events were monitored and recorded throughout the dosing period. After completion of the dosing period, the study sites had the option of either contacting subjects within 2–5 days or scheduling subjects for a follow-up office appointment to assess surgical healing, the occurrence or persistence of adverse events and medications taken. The primary efficacy end point was time to first perceptible pain relief. Secondary variables were time to meaningful PR, PI and PI differences, summed pain intensity differences (SPID) and total pain relief (TOTPAR) values at 2, 4 and 6 h and global evaluation.

### Statistical analysis

A sample size calculation for each study of approximately 500 subjects (200 subjects per aspirin treatment group and 100 subjects in the placebo group) provided 90% power to detect a treatment difference between FR-aspirin and R-aspirin for time to pain relief at a two-sided significance level of 0.05.

All hypotheses were tested at a two-sided significance level of 0.05. Efficacy variables were analyzed using all randomized subjects who took the study drug and who had at least one post-dose assessment on an efficacy parameter (intent to treat).

Time to first perceptible PR and meaningful PR were plotted using the Kaplan–Meier method and compared between treatment groups using the log-rank test. Median times to first perceptible PR and meaningful PR were calculated and presented.

Time-weighted SPIDs and TOTPARs were calculated for 6 h. An analysis of covariance (ANCOVA) model was used to compare means across treatment groups for the following secondary efficacy end points: SPID_0–2_, SPID_0–4_, SPID_0–6_, TOTPAR_0–2_, TOTPAR_0–4_, TOTPAR_0–6_ and PID at each post-dose time point. The ANCOVA model included treatment and center as the fixed effects and baseline pain as the covariate. In each analysis, adjusted least squares (LS) mean standard error and LS mean difference, along with 95% confidence intervals (CIs) of the difference, were obtained from the model.

## Results of Study 1: 500 mg aspirin tablet

Efficacy evaluations were made on the basis of the 514 patients included in the intent-to-treat (ITT) population for Study 1 (500 mg of aspirin). There were no group differences with respect to age, gender and baseline pain intensity (Table 1). The majority of patients were of younger age with a range of means of 22.4–22.7 years for the three treatment groups. At baseline, the overall mean PI score was 6.6 on the 11-point NRS. Approximately, 80% of patients in this study graded their baseline pain as moderate and 20% as severe. Approximately, three-quarters of the patients underwent removal of two impacted mandibular molars with a majority of patients having a total of three molars removed (Table 1).

**Table 1:** Summary of demographics and baseline characteristics

Study	Variable	Aspirin fast-release tablet	Aspirin regular tablet	Placebo
500 mg (Study 1)	ITT population	206	203	105
	Age, [years (mean + SD)]	22.4 (4.62)	22.7 (4.85)	22.5 (4.24)
	Gender ratio, male:female (%)	44.7:55.3	41.9:58.1	50.5:49.5
	11-point intensity [mean (SD)]	6.6 (1.14)	6.5 (1.07)	6.6 (1.17)
	Categorical pain intensity (%)			
	Moderate	80.6	80.3	79.0
	Severe	19.4	19.7	21.0
	Number of molars removed (%)			
	1	17.0	21.2	17.1
	2	82.0	78.3	79.0
	3	1.0	0.5	3.8
	Tooth sites (%)^a^			
	Left upper third molar	35.9	32.5	35.2
	Left lower third molar	43.7	38.9	41.0
	Right upper third molar	46.6	48.8	50.5
	Right lower third molar	57.8	59.1	60.0
	Impaction score (%)^a^			
	Erupted in tissue	23.3	26.6	24.8
	Broken soft tissue	10.2	13.8	21.0
	Partial bony impaction	38.3	32.5	29.5
	Full bony impaction	85.9	86.7	85.7
325 mg (Study 2)	ITT population	200	200	100
	Age, years [mean (SD)]	20.3 (3.33)	20.8 (4.05)	20.7 (3.57)
	Gender ratio, male:female (%)	31.5:68.5	41.5:58.5	34.0:66.0
	11-point intensity [mean (SD)]	7.3 (1.30)	7.4 (1.35)	7.5 (1.40)
	Categorical pain intensity (%)			
	Moderate	57.0	55.5	56.0
	Severe	43.0	44.5	44.0
	Number of molars removed (%)			
	1	6.0	3.0	8.0
	2	87.5	88.0	88.0
	3	0	0	0
	4	6.5	9.0	4.0
	Tooth sites (%)^a^			
	Left upper third molar	66.0	59.0	61.0
	Left lower third molar	69.0	65.0	68.0
	Right upper third molar	65.5	66.5	61.0
	Right lower third molar	68.5	73.5	66.0
	Impaction score (%)^a^			
	Erupted in tissue	9.0	8.5	14.0
	Broken soft tissue	5.5	8.5	8.0
	Partial bony impaction	45.0	50.0	41.0
	Full bony impaction	84.5	79.5	88.0

^a^Patients may have had more than one affected tooth site or impaction score

**Fig. 1:**
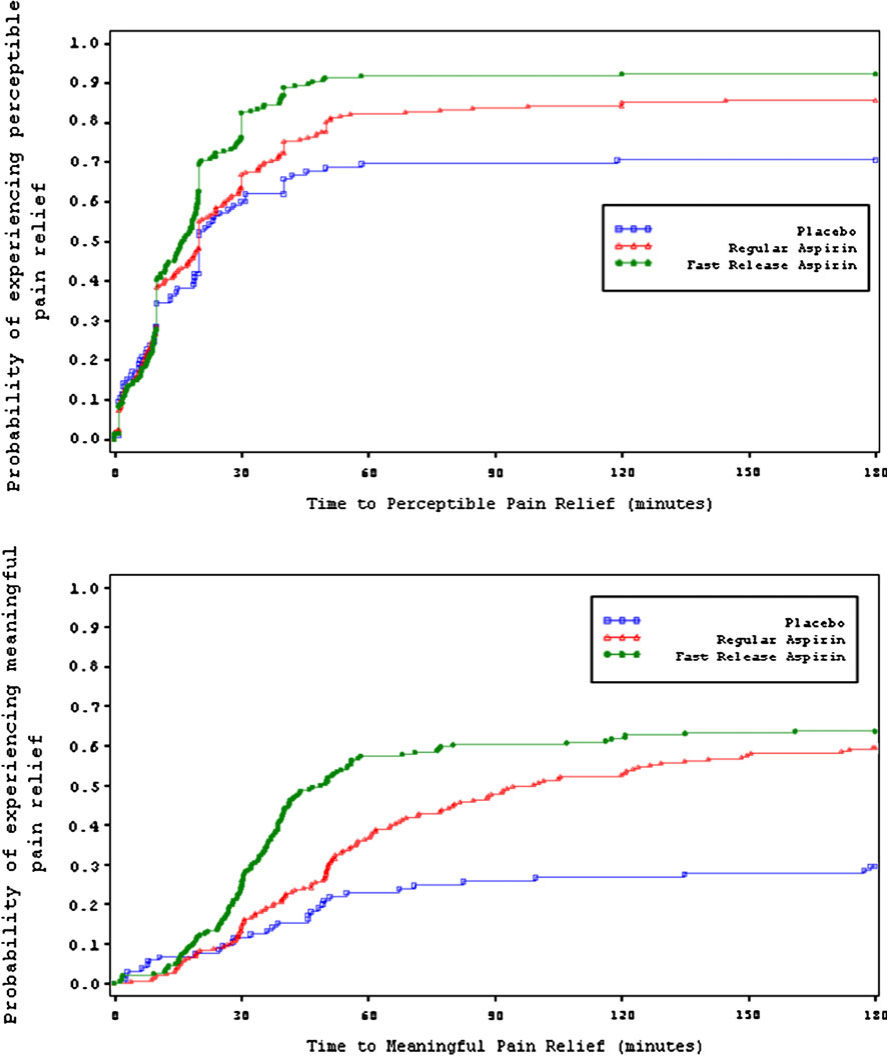
Kaplan–Meier plot of time to first perceptible and meaningful pain relief from aspirin 500 mg

**Fig. 2:**
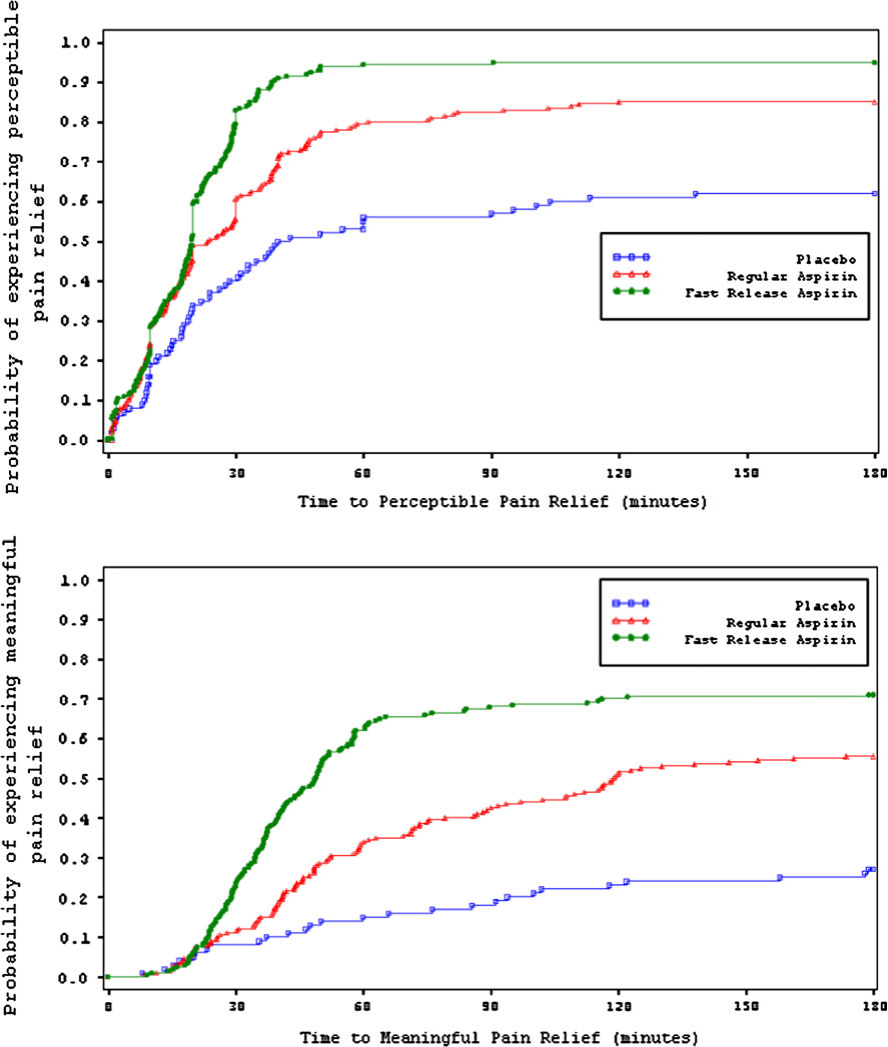
Kaplan–Meier plot of time to first perceptible and meaningful pain relief from aspirin 325 mg

Kaplan–Meier plots of time to first perceptible pain relief and meaningful pain relief showed a statistically significant difference between FR-aspirin tablets and R-aspirin tablets (*p* < 0.05). Both active treatments were significantly faster than placebo (*p* < 0.001) (Figs. 1, 2; Table 2). Median time to first perceptible pain relief was 16.3 min for FR-aspirin 500 mg and 20.0 min for R-aspirin 500 mg. Median time to meaningful pain relief was 49.4 min for FR-aspirin versus 99.2 min for R-aspirin. A higher percentage of patients in the FR-aspirin group achieved pain relief compared to R-aspirin and placebo (first perceptible pain relief: 92.2 vs. 85.7 vs. 70.5% and meaningful relief: 65.0 vs. 62.1 vs. 37.1%).

**Table 2:** Time to relief and efficacy results

Study	Variable	Aspirin fast-release tablet	Aspirin regular tablet	Placebo
500 mg (Study 1)	Median time to FPR, min (95% CI)	16.3 (12.2, 19.2)	20.0 (15.7, 23.4)	20.0 (18.9. 30.0)
	*p* value versus fast-release aspirin	–	0.004	<0.001
	*p* value versus regular aspirin	–	–	0.014
	% of subjects achieved PR	92.2	85.7	70.5
	% of subjects censored	7.8	14.3	29.5
	Median time to MR, min (95% CI)	49.4 (40.2, 57.7)	99.2 (77.0, 148.3)	NA (NA, NA)
	*p* value versus fast-release aspirin	–	0.038	<0.001
	*p* value versus regular aspirin	–	–	<0.001
	% of subjects achieved PR	65.0	62.1	37.1
	% of subjects censored	35.0	37.9	62.9
	SPID 0–2 h, mean (SD)	1.3 (1.5)	1.0 (1.5)	0.2 (1.3)
	*p* value versus fast-release aspirin	–	0.026	<0.001
	*p* value versus regular aspirin	–	–	<0.001
	SPID 0–4 h, mean (SD)	1.9 (3.2)	2.0 (3.4)	0.3 (3.2)
	*p* value versus fast-release aspirin	–	0.922	<0.001
	*p* value versus regular aspirin	–	–	<0.001
	SPID 0–6 h, mean (SD)	2.3 (4.7)	2.8 (5.3)	0.5 (5.2)
	*p* value versus fast-release aspirin	–	0.403	0.003
	*p* value versus regular aspirin	–	–	<0.001
	TOTPAR 0–2 h, mean (SD)	3.3 (2.2)	2.8 (2.2)	1.7 (2.0)
	*p* value versus fast-release aspirin	–	0.018	<0.001
	*p* value versus regular aspirin	–	–	<0.001
	TOTPAR 0–4 h, mean (SD)	5.7 (4.5)	5.6 (4.8)	3.5 (4.6)
	*p* value versus fast-release aspirin	–	0.882	<0.001
	*p* value versus regular aspirin	–	–	<0.001
	TOTPAR 0–6 h, mean (SD)	7.6 (6.6)	8.0 (7.5)	5.5 (7.6)
	*p* value versus fast-release aspirin	–	0.463	0.013
	*p* value versus regular aspirin	–	–	0.002
325 mg (Study 2)	Median Time to FPR, min (95% CI)	19.8 (18.2, 20.0)	23.7 (19.2, 30.0)	41.4 (30.7 103.9)
	*p* value versus fast-release aspirin	–	<0.001	<0.001
	*p* value versus regular aspirin	–	–	<0.001
	% of subjects achieved PR	95.0	85.0	62.0
	% of subjects censored	5.0	15.0	38.0
	Median time to MR, min (95% CI)	48.9 (41.8, 54.5)	119.2 (93.6, 192.3)	NA (NA, NA)
	*p* value versus fast-release aspirin	–	<0.001	<0.001
	*p* value versus regular aspirin	–	–	<0.001
	% of subjects achieved PR	72.5	59.0	30.0
	% of subjects censored	27.5	41.0	70.0
	SPID 0–2 h, mean (SD)	1.7 (1.6)	1.2 (1.5)	0.1 (1.2)
	*p* value versus fast-release aspirin	–	<0.001	<0.001
	*p* value versus regular aspirin	–	–	<0.001
	SPID 0–4 h, mean (SD)	2.8 (3.5)	2.7 (3.4)	0.3 (2.7)
	*p* value versus fast-release aspirin	–	0.668	<0.001
	*p* value versus regular aspirin	–	–	<0.001
	SPID 0–6 h, mean (SD)	3.5 (5.4)	3.8 (5.3)	0.6 (4.5)
	*p* value versus fast-release aspirin	–	0.631	<0.001
	*p* value versus regular aspirin	–	–	<0.001
	TOTPAR 0–2 h, mean (SD)	3.6 (2.1)	2.7 (2.1)	1.1 (1.4)
	*p* value versus fast-release aspirin	–	<0.001	<0.001
	*p* value versus regular aspirin	–	–	<0.001
	TOTPAR 0–4 h, mean (SD)	6.3 (4.8)	6.0 (4.8)	2.3 (3.4)
	*p* value versus fast-release aspirin	–	0.437	<0.001
	*p* value versus regular aspirin	–	–	<0.001
	TOTPAR 0–6 h, mean (SD)	8.5 (7.4)	8.6 (7.4)	3.7 (5.7)
	*p* value versus fast-release aspirin	–	0.875	<0.001
	*p* value versus regular aspirin	–	–	<0.001

*FPR* first perceptible pain relief, *MR* meaningful relief, *CI* confidence interval, *SPID* summed pain intensity differences, *TOTPAR* total pain relief. *NA* not available

The SPID and TOTPAR results were consistent with the greater efficacy for the FR-aspirin compared to the R-aspirin and placebo. At 2 h, differences between FR-aspirin and R-aspirin were significant [*p* = 0.026 (SPID) and *p* = 0.018 (TOTPAR)] and both actives were statistically superior compared with placebo (*p* < 0.001). For the 4- and 6-h measurements, SPID and TOTPAR for the FR-aspirin and R-aspirin were not different from each other, but both were statistically significantly greater than placebo (Table 2).

With respect to the global evaluation, the majority of subjects treated with FR-aspirin and R-aspirin rated their treatment as “fair” or better; while the majority of subjects treated with placebo rated their treatment as poor. More subjects treated with FR-aspirin assessed as very good or excellent (18.0 and 6.3%) compared with R-aspirin [13.8 and 5.4% (Table 3)].

**Table 3:** Global assessment of treatments

Study	Variable	Aspirin fast-release tablet (%)	Aspirin regular tablet (%)	Placebo (%)
500 mg (Study 1)	Poor	25.7	31.5	58.1
	Fair	26.2	20.7	13.3
	Good	22.3	28.1	19.0
	Very good	18.0	13.8	5.7
	Excellent	6.3	5.4	2.9
325 mg (Study 2)	Poor	19.5	29.0	60.0
	Fair	16.5	18.5	17.0
	Good	29.0	24.0	16.0
	Very good	22.0	19.5	5.0
	Excellent	12.5	8.0	0.0

Both active treatments were well tolerated. No serious adverse events were reported and no subject was discontinued due to adverse event in either 500 mg aspirin treatment group.

## Results of Study 2: 325 mg aspirin tablet

Efficacy evaluations were made on the basis of the 500 patients included in the intent-to-treat (ITT) population for Study 2 (325 mg aspirin). There were no group differences with respect to age, gender and baseline pain intensity (Table 1). The majority of patients were of younger age with a range of means of 20.3–20.8 years for the three treatment groups. At baseline, the overall mean PI score was 7.4 on the 11-point NRS. Approximately, 57% of patients graded moderate baseline pain and 43% severe baseline pain. Approximately, three-quarters of patients underwent removal of two impacted mandibular molars with a majority of patients having a total of four molars removed (Table 1).

Kaplan–Meier plots of time to first perceptible pain relief and meaningful pain relief showed a statistically significant difference between FR-aspirin tablets and R-aspirin tablets (*p* < 0.05). Both active treatments were significantly faster than placebo (*p* < 0.001) (Figs. 1, 2; Table 2). Median time to first perceptible pain relief was 19.8 min for FR-aspirin 325 mg and 23.7 min for R-aspirin 325 mg. In this study, median time to meaningful pain relief was 48.9 min for FR-aspirin versus 119.2 min for R-aspirin. A higher percentage of patients in the FR-aspirin group achieved pain relief compared to R-aspirin and placebo (first perceptible pain relief: 95.0 vs. 85.0 vs. 62.0% and meaningful relief: 72.5 vs. 59.0 vs. 30.0%).

The SPID and TOTPAR measurements showed the same consistent pattern as Study 1 for the comparisons between FR-aspirin, R-aspirin and placebo. At 2 h, differences between FR-aspirin and R-aspirin were significant (*p* < 0.001 SPID and TOTPAR) and both actives were statistically superior compared with placebo (*p* < 0.001). Similar to Study 1, for the 4- and 6-h SPID and TOTPAR measurements, the aspirin groups were not different; whereas both were statistically significantly greater than placebo (Table 2).

With respect to global evaluation, the majority of subjects treated with FR-aspirin and R-aspirin assessed as “fair” or better; while the majority of subjects treated with placebo assessed as “poor”. More subjects treated with FR-aspirin assessed as very good or excellent (22.0 and 12.5%) compared with R-aspirin [19.5 and 8.9% (Table 3)].

Both active treatments were well tolerated. In this study, there was one aspirin 325 mg subject in the R-aspirin group, who experienced a serious adverse event of gastritis that was considered not related to the study drug, and two subjects (1 in the R-aspirin group and 1 in the placebo group) who discontinued due to non-serious adverse events.

In both studies, the percentages of patients taking rescue medication (1,000 mg study: FR-aspirin = 57.8%, R-aspirin = 49.8%, placebo = 68.6% and 650 mg study: FR-aspirin = 59.0%, R-aspirin = 51.0%, placebo = 72.0%) and the median times to rescue medication (1,000 mg study: FR-aspirin = 252 min, R-aspirin > 360 min, placebo = 124 min and 650 mg study: FR-aspirin = 268 min, R-aspirin = 322 min, placebo = 105 min) were not statistically significantly different between active treatments, but both actives were significantly better than placebo (*p* < 0.001).

## Safety results

Adverse events of the individual studies are shown in Table 4. In Study 1, between 15 and 22% of subjects experienced at least one adverse event; in Study 2, between 14 and 18%. Gastrointestinal adverse events appeared between 4 and 8% in Study 1 and between 5 and 8% in Study 2. The most common GI adverse event was nausea.

**Table 4:** Treatment emergent adverse events of individual studies with aspirin frequencies >1% [number (percentages)]

Study	Study 1 aspirin 1,000 mg			Study 2 aspirin 650 mg		
Variable	Aspirin fast-release tablet	Aspirin regular tablet	Placebo	Aspirin fast-release tablet	Aspirin regular tablet	Placebo
Total number of patients treated	206	203	105	200	200	100
Total number of adverse events	50	36	36	41	39	23
Number of subjects with at least one adverse event	38 (18.4%)	31 (15.3%)	23 (21.9%)	29 (14.5%)	30 (15.0%)	18 (18.0%)
Gastrointestinal disorders	10 (4.9%)	8 (3.9%)	8 (7.6%)	13 (6.5%)	10 (5.0%)	8 (8.0%)
Nausea	8 (3.9%)	6 (3.0%)	5 (4.8%)	9 (4.5%)	5 (2.5%)	6 (6.0%)
Vomiting	5 (2.4%)	1 (0.5%)	3 (2.9%)	3 (1.5%)	2 (1.0%)	1 (1.0%)
Nervous system disorders	12 (5.8%)	16 (7.9%)	13 (12.4%)	5 (2.5%)	11 (5.5%)	5 (5.0%)
Dizziness	4 (1.9%)	2 (1.0%)	2 (1.9%)	4 (2.0%)	3 (1.5%)	3 (3.0%)
Headache	8 (3.9%)	14 (6.9%)	9 (8.6%)	1 (0.5%)	8 (4.0%)	1 (1.0%)
Infections and infestations	13 (6.3%)	9 (4.4%)	5 (4.8%)	9 (4.5%)	8 (4.0%)	3 (3.0%)
Alveolar osteitis	12 (5.8%)	9 (4.4%)	5 (4.8%)	6 (3.0%)	6 (3.0%)	3 (3.0%)

## Discussion

If an analgesic works fast, the consumers get the relief they seek and then there is less likelihood of taking additional medication that can result in unwanted side effects. In the recent past, several OTC analgesics were introduced as softgel or liquigel formulations with the putative advantage of a faster onset of action (Doyle et al. 2002). Interestingly, to our knowledge, none of these analgesics has ever directly compared the new formulation to the original formulation in a double-blinded efficacy study. These drugs rely on dissolution and pharmacokinetic data to justify their putative onset advantage; but this is not necessarily directly correlated into a clinical advantage. In the two studies presented, the efficacy data clearly demonstrate that the new formulation of aspirin has a faster onset of action at both the 650 mg and 1,000 mg doses. For the aspirin 650 mg dose, the median time to meaningful relief was 70.3 min faster than regular aspirin, and for the 1,000 mg aspirin dose, the median time to meaningful relief was 49.8 min faster than regular aspirin. From a clinical standpoint, this is a very relevant advantage and was accomplished without the addition of any active adjuvant such as caffeine. This advanced aspirin formulation was accomplished by incorporating an effervescent component (sodium carbonate) and micronized active ingredient. Unlike a softgel or liquigel formulation, this aspirin formulation retains the advantage of still being a tablet.

Furthermore, the overall efficacy of the FR-aspirin tablet was sustained as shown by the non-significant differences between FR-aspirin and R-aspirin for the SPID- and TOTPAR measurements and the use of rescue medication over the 6-h observation period.

The dental impaction pain model was chosen for these studies because of its proven validity, reliability and sensitivity. The results of these two studies are consistent with previous dental studies and there was a strong correlation between the improved pharmacokinetics of the new aspirin formulation and clinical efficacy.

It can be postulated that the minor outcome differences between the two studies can be explained by the expected variability between the two different study sites. Furthermore, distribution of baseline pain intensity was different in the two studies. However, the results of the two studies were remarkably consistent and supportive of the faster onset for the FR-aspirin formulations.

## Conclusions

The two efficacy studies described in this paper clearly demonstrate that the onset of analgesic efficacy is improved by adding an effervescent component (sodium carbonate) and micronized active ingredient to the aspirin formulation. The enhanced onset did not adversely impact either the peak effect or duration of effect compared to regular aspirin. In addition, the side effect data from these single dose studies did not indicate any change in the side effect profile. The initial findings of significantly improved dissolution and pharmacokinetic profiles for this new rapidly acting aspirin formulation were confirmed in these two well-controlled efficacy studies.
